# Ankle manual therapy for individuals with post-acute ankle sprains: description of a randomized, placebo-controlled clinical trial

**DOI:** 10.1186/1472-6882-10-59

**Published:** 2010-10-19

**Authors:** Todd E Davenport, Kornelia Kulig, Beth E Fisher

**Affiliations:** 1 Department of Physical Therapy, Thomas J. Long School of Pharmacy and Health Sciences, University of the Pacific, Stockton, California, USA; 2 Musculoskeletal Biomechanics Research Laboratory, Division of Biokinesiology and Physical Therapy at the Herman Ostrow School of Dentistry, University of Southern California, Los Angeles, California, USA; 3 Neuroplasticity and Imaging Laboratory, Division of Biokinesiology and Physical Therapy at the Herman Ostrow School of Dentistry, University of Southern California, Los Angeles, California, USA

## Abstract

**Background:**

Ankle sprains are common within the general population and can result in prolonged disablement. Limited talocrural dorsiflexion range of motion (DF ROM) is a common consequence of ankle sprain. Limited talocrural DF ROM may contribute to persistent symptoms, disability, and an elevated risk for re-injury. As a result, many health care practitioners use hands-on passive procedures with the intention of improving talocrural joint DF ROM in individuals following ankle sprains. Dosage of passive hands-on procedures involves a continuum of treatment speeds. Recent evidence suggests both slow- and fast-speed treatments may be effective to address disablement following ankle sprains. However, these interventions have yet to be longitudinally compared against a placebo study condition.

**Methods/Design:**

We developed a randomized, placebo-controlled clinical trial designed to test the hypotheses that hands-on treatment procedures administered to individuals following ankle sprains during the post-acute injury period can improve short-, intermediate-, and long-term disablement, as well as reduce the risk for re-injury.

**Discussion:**

This study is designed to measure the clinical effects of hands-on passive stretching treatment procedures directed to the talocrural joint that vary in treatment speed during the post-acute injury period, compared to hands-on placebo control intervention.

**Trial Registration:**

http://www.clinicaltrials.gov identifier NCT00888498.

## Background

Ankle sprains are the most common injury to the ankle joint, accounting for up to 2 million injuries per year [1]. Annual incidence is estimated at 52.7 per 10,000 individuals [2]. Ankle injuries are very common in younger and active individuals, second only to the knee in the annual incidence of lower extremity sports-related injuries [3,4]. Among high school athletes in the United States, sprains account for 50% of all lower extremity injuries [5] with the ankle joint most commonly affected [5-7]. In one sample of individuals with non-athletic mechanisms of injury, sprains accounted for over 40% of reported injuries with the ankle joint also most commonly affected [8]. Certain sports and work activities may result in an even higher incidence and risk for injury [9-15]. Ankle sprains are clinically significant because they result in a substantial number of missed work days [8] and participation in sports activity [3,5], as well as potential early arthritic changes in the talocrural joint [16].

Lateral ankle and midfoot injuries account for 80-85% of all sprains [17,18]. The most common mechanism of injury for ankle sprains involves plantarflexion and inversion of the ankle and foot, which places excessive load on the anterior talofibular ligament. With failure of this ligament, secondary restrain to inversion occurs by way of the calcaneofibular and posterior talofibular ligaments, placing them at similar risk for injury. The anteromedial joint capsule and anterior fibers of the deltoid ligament may be secondarily injured due to excessive eversion of the ankle and foot that may occur during recoil from maximal inversion. Ankle sprains are assigned grades I to III, ordered from least severe to most severe ligament damage.

Although the prognosis for functional recovery following ankle sprain typically includes a rapid reduction in disablement within the first 2 weeks after injury [19], recent studies indicate a subgroup of individuals appears predisposed to continued pain, functional deficits, and prolonged risk for additional re-injury [19-26]. For example, Gerber and colleagues [17] reported 95% of their sample of injured military cadets returned to normal activities within 6 weeks, although 55% reported residual functional deficits. At 6-month follow-up, 40% of the sample continued to note functional deficits compared to their pre-injury status. Other studies have corroborated reports of prolonged functional deficits and risk for re-injury as long as 18 months to 3 years following the initial injury [19,20,26]. The incidence and prolonged disability associated with ankle sprains requires identification of optimal approaches to clinical management.

One reason for continued pain and elevated risk for re-injury may be limited ankle joint mobility. Several studies have identified limited talocrural joint dorsiflexion range of motion (DF ROM) as an important predisposing factor to ankle sprains [27-29]. Significant short-term loss of ankle range of motion also has been documented as a response to injury in persons who sustained a recent ankle sprain [30]. Along these lines, Denegar and colleagues [31] established a significant loss of posterior talar glide in individuals with unilateral ankle sprains, although a significant difference in talocrural DF ROM between involved and uninvolved sides was not noted. One important implication of studies that have identified talocrural DF ROM deficits in individuals following ankle sprains is that perhaps intervention to improve talocrural DF ROM should correspondingly reduce ankle pain, disability, and risk for re-injury following ankle sprains.

Many clinicians use hands-on passive stretching techniques to improve limited joint mobility, such as ankle DF ROM. Hands-on passive stretching techniques, including mobilization and manipulation, vary on a dosage continuum involving the speed of iatrogenic force application. Mobilization typically involves a slow and sustained application of iatrogenic force, while manipulation is characterized by a rapid application of iatrogenic force. Despite the intuitive appeal of applying mobilization and manipulation to promote parallel improvements in talocrural DF ROM and functioning in individuals following ankle sprains, this idea has been the focus of relatively few prospective studies [32]. Pellow and Brantingham [33] were among the first to report reduced pain and improved function in individuals with ankle sprains receiving an ankle mortise distraction technique. Whitman and colleagues [34] reported rapid functional improvement after talocrural manipulation in a single case study of a competitive volleyball player with a mild unilateral ankle sprain. More recently, Whitman and coworkers [35] documented favourable clinical outcomes in approximately 75% of their sample with post-acute ankle sprains following 2 sessions of mobilization and manipulation directed at joints distal to the knee.

Although initial results are promising, various methodological issues in existing studies challenge our collective understanding of the effectiveness of mobilization and manipulation on clinical outcomes in individuals with ankle sprains. The effect attributable to placebo manual stimulation and speed of iatrogenic force application during hands-on treatment remains unclear. Also, prior studies have not conducted longitudinal assessments over sufficient durations to adequately evaluate the effectiveness of mobilization/manipulation to reduce the risk for re-injury. Thus, the purpose of this paper is to describe the methodology of a randomized, placebo-controlled clinical trial to evaluate the effect of mobilization, manipulation, and hands-on placebo treatment of the talocrural joint on short-, intermediate-, and long-term clinical outcomes in individual with post-acute ankle sprains.

## Methods/Design

### Subjects

Patients with post-acute ankle sprains will be recruited to participate in this study from multiple clinical centers across the United States. The Institutional Review Boards of the University of the Pacific (Stockton, CA, USA) and Des Moines University (Des Moines, IA, USA) approved this study protocol. This study protocol is registered with http://www.clinicaltrials.gov (NCT00888498).

Inclusion criteria for this study are age 16-60 years, onset of ankle sprain at least 2 weeks prior to enrollment, and Foot and Ankle Ability Measure Activities of Daily Living (FAAM ADL) subscale score less than or equal to 67 points (ie, respondent indicates ≥20% disability). Exclusion criteria involve current assisted ambulation (eg, cane or crutches); inability to bear weight through the affected extremity immediately after injury with tenderness to palpation of the medial and lateral malleolar zones, styloid process of the 5^th ^metatarsal, and navicular [36]; positive anterior drawer or inversion stress maneuver suggesting ligamentous laxity [37-39]; volume of the affected limb greater than 10% of the unaffected limb per water displacement volumetry [40]; previous history of ligament or bony reconstructive surgery to the ankle and foot; concomitant injury to other lower extremity joints; and inability to comply with the treatment protocol.

**Figure 1:**
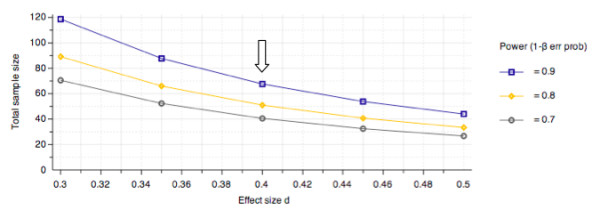
**Group size vs. effect size plot given d = .4 (arrow), β ≥ .80, and α = .05 for a 2-sample t-test**. Optimal sample size occurs at n = 52 per group. Adding an additional 20% to group size to account for potential drop-outs, group size for this study was estimated at n = 63.

A total of 189 subjects will be recruited (n = 63 per group). A group size of 52 subjects will provide 80% statistical power to detect an 8-point (9.5%) difference in FAAM ADL subscale score based on Martin and colleagues' data[41], with additional subjects recruited to account for a 20% dropout rate [42]. This sample size ensures the study is powered to detect a statistically and clinically significant difference between groups using a 2-sample t-test (Figure 1).

### Pre-Participation Screening Measures

#### Foot and Ankle Ability Measure (FAAM)

The FAAM is a self-report questionnaire consisting of a 21-item ADL subscale and 8-item Sports subscale, in addition to a global rating of self-perceived foot function from 0-100% [41]. This inventory demonstrates satisfactory internal consistency, validity, and score stability. Test-retest validity is satisfactory for use in research designs involving repeated measures (ICC_2,1 _= .87-.89). Minimum clinically important change scores have been identified for the ADL subscale (8 points) and Sports subscale (9 points) with respect to patient global rating of change.

#### Anterior drawer test

The anterior drawer test is a manual assessment of the integrity of the anterior talofibular ligament (ATFL) [43]. It is performed with the patient in supine hook-lying position to relax the plantaflexor muscle group, and the ankle of interest plantarflexed 10-15 degrees [44]. The evaluating therapist stabilizes the tibia and fibula while translating the calcaneus and talus anteriorly. A positive test involves the perception of excessive movement by the investigator or the appearance skin dimpling on the anterolateral ankle [45]. This test demonstrates diagnostic specificity to detect rupture of the ATFL, and as little as 2 mm of excessive excursion may be detected manually [37,39].

#### Inversion stress maneuver

The inversion stress test is intended to elicit symptoms or excessive mobility due to calcaneofibular ligament rupture [38]. It is performed with the subject in supine. The evaluating therapist stabilizes the affected leg, and moves the calcaneus into inversion and adduction, with the foot positioned neutrally in the sagittal plane.

#### Volumetric measurement of the foot, ankle, and lower leg

Water displacement volumetry will be used to assess for the presence of clinically significant ankle edema, which is operationally defined as ≤10% difference in left and right limb volume in this study. In this test, the subject will first place their uninvolved foot in a 32.5 × 12.5 × 22.5 cm clear acrylic box containing a known volume of water. As the subject's foot enters the box, water will flow from a spout in a box into a graduated cylinder. Measurement of displaced water will be completed and then the displaced water will be discarded. The graduated cylinder will be dried thoroughly and replaced beneath the box spout. The subject will then place their involved foot into the box, and the volume of displaced water will be measured. This volume represents the difference in volumes between involved and uninvolved sides. The residual volume of water displaced by the involved side will then be divided by the volume of water displaced by the uninvolved side. Water displacement volumetry demonstrates strong intra-tester reliability (ICC_2,1_=.99) and inter-tester reliability (ICC_2,1_=.93-.97) [46,47]. The 10% inter-limb difference criteria adopted for this study exceeds the margin of diurnal variability in water displacement measurements [40].

### Baseline Measures

After pre-participation screening is completed and informed consent is obtained, baseline measures will be collected on the same day.

#### Numeric Pain Rating Scale (NPRS) and Pain Diagram

The 11-point NPRS will be used to structure 3 measurements of subjects' pain intensity, including the levels at best in the past 24 hours, at worst in the past 24 hours, and the current level of pain. The NPRS ranges from "No Pain" and "Worst Imaginable Pain." NPRS measurements are reliable and valid for use in clinical trials [48]. A pain diagram will be used to record the location and nature of a patient's ankle and foot symptoms by drawing it on the diagram of a human figure.

#### Fear Avoidance Beliefs Questionnaire (FABQ)

The FABQ is a questionnaire intended to quantify the level of fear of pain during various activities in individuals with lower back pain [49]. It consists of a Physical Activity subscale (4 items) and Work subscale (7 items) and 5 distractor items. The subscales of the FABQ demonstrate high test-retest reliability (ICC_2,1_=.77 and .90, respectively) [50]. Each subscale has been demonstrated to be significantly predictive of current and future disability in individuals with acute and chronic lower back pain [49,51,52]. Wording will be changed to reference lower extremity disability for purposes of this study. Preliminary data regarding clinimetric properties of the modified FABQ will be collected during this study in order to conduct future validation analyses.

#### Lower Extremity Self Efficacy Scale (LExSES)

The LExSES is a 20-item questionnaire intended to measure a subjects' self-efficacy related to lower extremity function. It consists of 3 subscales of patient self-efficacy, including functional and self-regulatory abilities, and exercise performance self-efficacy. Preliminary data regarding clinimetric properties of the LExSES also will be collected during this study in order to conduct future validation analyses.

#### Positive and Negative Affect Scale (PANAS)

The PANAS is an inventory aimed at assessing a patients' mood. It consists of two checklists, each containing 10 items [53]. Patients are requested to rate each item to describe their mood in the timeframe provided using a 5-point Likert scale. The response scale ranges from 1 "Very slightly or not at all" to 5 "Extremely." The PANAS shows high internal consistency, test-retest reliability, and sensitivity to change.

#### Patient Global Rating of Change (GROC)

The GROC is a single item 15-point scale intended to measure a subject's general perception regarding response to treatment [54]. Responses range from "A very great deal better" to "A very great deal worse," with "About the same" serving as the center of the scale. GROC is a common scale used in studies that assess health-related quality of life due to its simplicity and face validity.

#### Lower extremity ROM

Hip flexion with knee flexed, internal rotation, external rotation; knee extension; subtalar inversion and eversion; and ankle plantarflexion and dorsiflexion passive ROM will be measured with a standard goniometer by the evaluating therapist using previously described techniques that demonstrate adequate validity and reliability [55-58]. An inclinometer will be used to measure hip extension range of motion in a Thomas test position with both the knee flexed and extended [59,60] and the inclinometer placed just proximal to the patella and parallel with the long axis of the thigh.

#### Lower extremity manual muscle testing (MMT)

MMT of the gluteus medius, gluteus maximus, quadriceps, tibialis posterior, tibialis anterior, peroneus longus/brevis (group), and gastocnemius will be completed according to established procedures and grading scheme [59]. Pain and inability to maintain the test position at a given level of resistance will be included as criteria to discontinue the tests. These tests retain adequate validity and intra-rater reliability to detect muscle weakness, although they may demonstrate ceiling effects that depend on examiner characteristics [61].

#### Star balance excursion test (SBET)

The SBET is a clinical test of dynamic balance [62-65]. Subjects will assume unilateral stance in the center of a grid marked circumferentially in 45-degree increments. After a learning trial consisting of 6 repetitions in each of the 8 test directions [63], subjects will complete 3 repetitions of single limb squat reach. Two trials will be completed: 1 trial each with the subject standing on the affected and unaffected limbs. Test directions include anterior, lateral, anterolateral, posterolateral, posterior, medial, anteromedial, and posteromedial. The evaluating therapist will record the distance achieved between the stance toe and heel of the reaching extremity for 3 repetitions in each direction. Fifteen seconds of rest will be provided between trials. Repetitions will be excluded if the subject (1) is unable to maintain weightbearing during the trial; (2) lifts the stance foot; (3) loses balance; or (4) does not maintain the hold or start positions for 1 second. This test demonstrates good reliability (ICC_2,1 _= .67-.97), and demonstrates discriminative validity between non-disabled individuals and patients with chronic ankle instability [64,66].

### Randomization and blinding

After completing pre-participation screening and baseline measures by a standardized licensed physical therapist (evaluating therapist), each subject will receive both an identification number and a unique random number indicating treatment group assignment. Numbers indicating group assignments will be concealed in a privacy envelope. A second licensed physical therapist blinded to the baseline examination (treating therapist) will open the proper randomization envelope that corresponds to the patient's unique identification number. The treating therapist will record the subject's group assignment on a standardized form. It will be impossible to blind the subject and treating therapist to treatment assignment. However, the evaluating therapist will remain blind to the subject's treatment assignment. Patients will be instructed to conceal their group assignment from the evaluating therapist.

### Experimental groups

**Figure 2:**
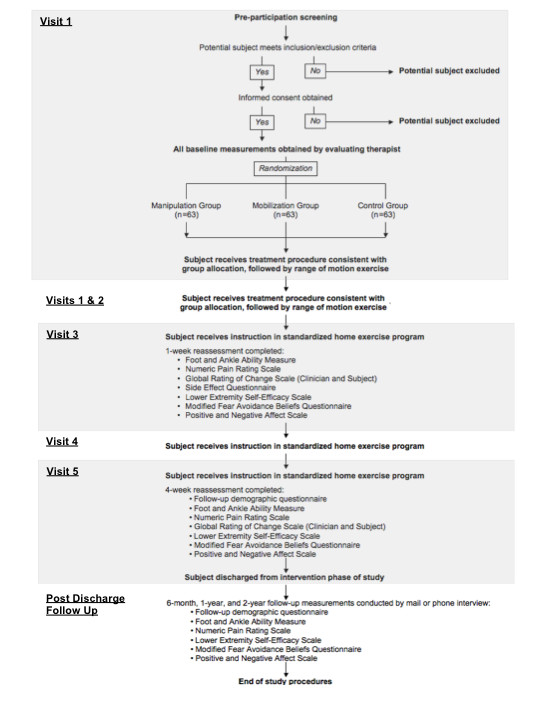
**Flow chart for subject screening, pre-intervention measurement, intervention, and post-intervention measurements**.

**Figure 3:**
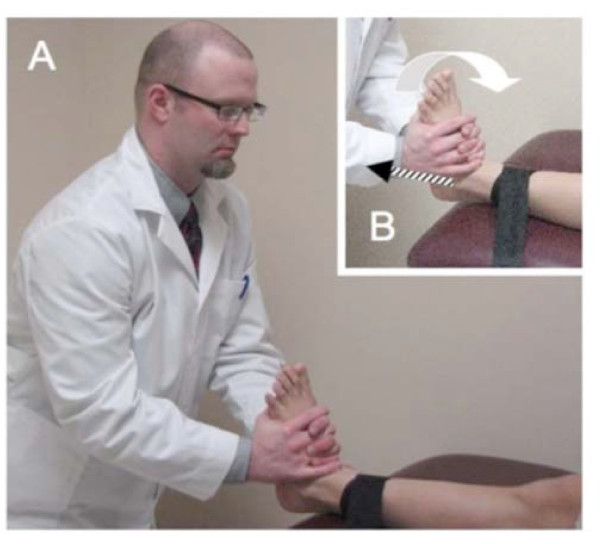
**Ankle high-velocity low-amplitude, slow velocity, and control interventions under study**. With the subject in a supine position on a treatment table and the lower extremity of interest stabilized to the table with a belt (A), the treating investigator will grasp the foot of interested with the thenar eminences on the foot's plantar surface (B) and induce passive dorsiflexion to end range (B; open arrow). Iatrogenic force will be provided along the long axis of the tibia in the intervention groups. (B; hatched line) In the control group, the treating investigator will maintain passive dorsiflexion (B; open arrow) for the duration of 1 deep inhalation and exhalation by the subject rather than induce an iatrogenic force.

Subjects will be randomized into 3 groups after pre-participation screening is completed, informed consent is obtained, and baseline measures are recorded (Figure 2). The first group (n = 63) will receive talocrural traction manipulation (Figure 3). With the subject in a seated position on a treatment table and the lower extremity of interest stabilized to the table with a belt, the standardized treating therapist will grasp the foot of interest with the thenar eminences on the foot's plantar surface. A thrust will be delivered parallel to the long axis of the subject's lower leg after the treating therapist induces passive ankle dorsiflexion to end range. The second group (n = 63) will receive talocrural traction mobilization. Traction will be delivered to the talocrural joint at the treating therapist's second perception of tissue resistance in 3 bouts of 30-second holds, separated by 10 seconds of rest. The third group (n = 63) will receive the manual therapy control intervention. This will consist of the same patient and clinician preparation for the mobilization/manipulation techniques. However, the treating therapist will maintain passive DF ROM for the duration of 1 deep inhalation and exhalation by the subject. Following the procedure, all subjects will receive range of motion exercises involving 10 clockwise ankle and foot circles within a symptom-free range of motion. Subjects will be requested to complete this exercise 4 times daily for 10 repetitions each session.

### Treatment schedule

All subjects will attend 5 treatment sessions as part of this study, including 2 sessions during the first week and 1 session per week for the next 3 consecutive weeks. All subjects will be scheduled for the first treatment session within 3 days of the baseline examination. In all treatment groups, the first 2 sessions will begin with delivery of the ankle manual therapy technique followed by the range of motion exercise described above. The final 3 sessions will involve instruction in a standardized strengthening program. The treating therapist will document daily treatment on a standardized form, as well as their subjective impression of the subject's improvement at 4 weeks after enrolment. Subjects will be requested to document home program compliance on a standardized log, and advised to refrain from new activities during the study. Clinicians and subjects will be free to pursue their treatments of choice after the 4-week measurements are obtained.

### Post-treatment measures and schedule

The FAAM, NPRS, FABQ, PANAS, LExSES, and a side effect questionnaire will be administered at 1 week following enrolment. The demographic questionnaire, FAAM, NPRS, FABQ, PANAS, and LExSES will be administered at 4 weeks following enrolment, and mailed to subjects to complete at 6 months, 1 year, and 2 years following enrolment.

### Data Analysis

The primary dependent variable for this study is FAAM score at 1 week, 4 weeks, 6 months, 1 year, and 2 years. Secondary dependent variables include NPRS, modified FABQ, PANAS, LExSES, and GROC scores at 1 week, 4 weeks, 6 months, 1 year, and 2 years. The independent variable is the manual therapy intervention condition. All other outcome measures will be assessed as potential covariates of treatment success, which is defined as the achievement of minimum clinically important change score on the FAAM for this study.

Descriptive statistics will be calculated for the data set, including frequencies, means, and standard deviations. Between and within-group comparisons will be made for outcome measurements at 4 weeks, 6 months, 1 year, and 2 years post-enrolment. Analysis of variance with appropriate post-hoc pairwise comparisons will be used to assess between-groups differences for continuous data. Statistical significance of between-group differences in categorical data will be assessed with chi square analysis. Pearson correlations and multivariate linear regression analyses will be performed to determine statistical significance of associations with treatment success and failure. An intention-to-treat analysis will be performed for subjects who drop out of the study after enrolment but prior to completion of all study procedures. Statistical significance of inferential statistical tests will be accepted at α≤.05.

## Discussion

This study protocol describes a randomized, placebo-controlled clinical trial that measures the short-, intermediate-, and long-term effects of talocrural joint mobilization, manipulation, and placebo treatment for individuals with post-acute ankle sprains. Hands-on treatments that improve ankle DF ROM are hypothesized to cause related improvement in disability and mitigation of re-injury risks. We believe the study designed in this paper addresses methodological deficiencies in the manual therapy literature to date that interfere with our optimal understanding of best practices related to mobilization/manipulation in individuals with post-acute ankle sprains. Specifically, inclusion of a hands-on placebo control should allow for differentiation of generalized clinical benefit from manual stimulation and clinician attention versus iatrogenic force application. In addition, the length of follow-up measurements should allow for adequate estimation of the potential longitudinal effects of mobilization/manipulation during the post-acute injury period on re-injury rate. The methodological characteristics of this study should help generate high quality data that will further our collective understanding of best practices in manual therapy.

## Abbreviations

ADL: activities of daily living; ATFL: anterior talofibular ligament; DF: dorsiflexion; FAAM: Foot and Ankle Ability Measure; FABQ: Fear Avoidance Beliefs Questionnaire; GROC: Global rating of change; ICC_2,1_: Intraclass correlation coefficient - formula 2,1; LExSES: Lower Extremity Self Efficacy Scale; MMT: manual muscle test; NPRS: numeric pain rating scale; PANAS: Positive and Negative Affect Scale; RCT: randomized clinical trial; RICE: rest, ice, compression, and elevation; ROM: range of motion

## Competing interests

The authors declare that they have no competing interests.

## Authors' contributions

TED, KK, and BEF contributed to the design of this study protocol. TED provided research funding. All authors read and approved the final manuscript.

## Pre-publication history

The pre-publication history for this paper can be accessed here:

http://www.biomedcentral.com/1472-6882/10/59/prepub

## References

[B1] Beynnon BD Renstrom PA Alosa DM Baumhauer JF Vacek PM Ankle ligament injury risk factors: a prospective study of college athletes J Orthop Res 2001 19 2 213 220 10.1016/S0736-0266(00)90004-4 11347693

[B2] Bridgman SA Clement D Downing A Walley G Phair I Maffulli N Population based epidemiology of ankle sprains attending accident and emergency units in the West Midlands of England, and a survey of UK practice for severe ankle sprains Emerg Med J 2003 20 6 508 510 10.1136/emj.20.6.508 14623833 PMC1726220

[B3] Bahr R Reeser JC Injuries among world-class professional beach volleyball players. The Federation Internationale de Volleyball beach volleyball injury study Am J Sports Med 2003 31 1 119 125 12531768 10.1177/03635465030310010401

[B4] Fong DT Hong Y Chan L Yung PH Chan K A systematic review on ankle injury and ankle sprain in sports Sports Medicine 2006 37 1 73 94 10.2165/00007256-200737010-00006 17190537

[B5] Fernandez WG Yard EE Comstock RD Epidemiology of lower extremity injuries among U.S. high school athletes Acad Emerg Med 2007 14 7 641 645 17513688 10.1197/j.aem.2007.03.1354

[B6] Fordham S Garbutt G Lopes P Epidemiology of injuries in adventure racing athletes Br J Sports Med 2004 38 3 300 303 10.1136/bjsm.2002.003350 15155432 PMC1724844

[B7] Jacobson BH Hubbard M Redus B Price S Palmer T Purdie R Altena T An assessment of high school cheerleading: injury distribution, frequency, and associated factors J Orthop Sports Phys Ther 2004 34 5 261 265 15189018 10.2519/jospt.2004.34.5.261

[B8] Grimm DJ Fallat L Injuries of the foot and ankle in occupational medicine: a 1-year study J Foot Ankle Surg 1999 38 2 102 108 10.1016/S1067-2516(99)80020-9 10334696

[B9] Bahr R Bahr IA Incidence of acute volleyball injuries: a prospective cohort study of injury mechanisms and risk factors Scand J Med Sci Sports 1997 7 3 166 171 10.1111/j.1600-0838.1997.tb00134.x 9200321

[B10] Hickey GJ Fricker PA McDonald WA Injuries of young elite female basketball players over a six-year period Clin J Sport Med 1997 7 4 252 256 10.1097/00042752-199710000-00002 9397323

[B11] Kirkpatrick DP Hunter RE Janes PC Mastrangelo J Nicholas RA The snowboarder's foot and ankle Am J Sports Med 1998 26 2 271 277 9548123 10.1177/03635465980260021901

[B12] Kujala UM Nylund T Taimela S Acute injuries in orienteerers Int J Sports Med 1995 16 2 122 125 10.1055/s-2007-972977 7751075

[B13] Linko PE Blomberg HK Frilander HM Orienteering competition injuries: injuries incurred in the Finnish Jukola and Venla relay competitions Br J Sports Med 1997 31 3 205 208 10.1136/bjsm.31.3.205 9298554 PMC1332519

[B14] McGaughey I Sullivan P The epidemiology of knee and ankle injuries on Macquarie Island Injury 2003 34 11 842 846 10.1016/S0020-1383(03)00032-9 14580818

[B15] Verhagen EA Van der Beek AJ Bouter LM Bahr RM Van Mechelen W A one season prospective cohort study of volleyball injuries Br J Sports Med 2004 38 4 477 481 10.1136/bjsm.2003.005785 15273190 PMC1724865

[B16] Larsen E Jensen PK Jensen PR Long-term outcome of knee and ankle injuries in elite football Scand J Med Sci Sports 1999 9 5 285 289 10.1111/j.1600-0838.1999.tb00247.x 10512209

[B17] Gerber JP Williams GN Scoville CR Arciero RA Taylor DC Persistent disability associated with ankle sprains: a prospective examination of an athletic population Foot Ankle Int 1998 19 10 653 660 9801078 10.1177/107110079801901002

[B18] Holmer P Sondergaard L Konradsen L Nielsen PT Jorgensen LN Epidemiology of sprains in the lateral ankle and foot Foot Ankle Int 1994 15 2 72 74 7981804 10.1177/107110079401500204

[B19] van Rijn RM van Os AG Bernsen RM Luijsterburg PA Koes BW Bierma-Zeinstra SM What is the clinical course of acute ankle sprains? A systematic literature review Am J Med 2008 121 4 324 331 e326 10.1016/j.amjmed.2007.11.018 18374692

[B20] Braun BL Effects of ankle sprain in a general clinic population 6 to 18 months after medical evaluation Arch Fam Med 1999 8 2 143 148 10.1001/archfami.8.2.143 10101985

[B21] Garrick JG Requa RK The epidemiology of foot and ankle injuries in sports Clin Podiatr Med Surg 1989 6 3 629 637 2568882

[B22] Kofotolis ND Kellis E Vlachopoulos SP Ankle sprain injuries and risk factors in amateur soccer players during a 2-year period Am J Sports Med 2007 35 3 458 466 10.1177/0363546506294857 17218660

[B23] McKay GD Goldie PA Payne WR Oakes BW Ankle injuries in basketball: injury rate and risk factors Br J Sports Med 2001 35 2 103 108 10.1136/bjsm.35.2.103 11273971 PMC1724316

[B24] Milgrom C Shlamkovitch N Finestone A Eldad A Laor A Danon YL Lavie O Wosk J Simkin A Risk factors for lateral ankle sprain: a prospective study among military recruits Foot Ankle 1991 12 1 26 30 1959831 10.1177/107110079101200105

[B25] Tyler TF McHugh MP Mirabella MR Mullaney MJ Nicholas SJ Risk factors for noncontact ankle sprains in high school football players: the role of previous ankle sprains and body mass index Am J Sports Med 2006 34 3 471 475 10.1177/0363546505280429 16260467

[B26] Anandacoomarasamy A Barnsley L Long term outcomes of inversion ankle injuries Br J Sports Med 2005 39 3 e14 discussion e14 10.1136/bjsm.2004.011676 15728682 PMC1725165

[B27] de Noronha M Refshauge KM Herbert RD Kilbreath SL Hertel J Do voluntary strength, proprioception, range of motion, or postural sway predict occurrence of lateral ankle sprain? Br J Sports Med 2006 40 10 824 828 discussion 828 10.1136/bjsm.2006.029645 16920769 PMC2465053

[B28] Pope R Herbert R Kirwan J Effects of ankle dorsiflexion range and pre-exercise calf muscle stretching on injury risk in Army recruits Aust J Physiother 1998 44 3 165 172 11676730 10.1016/s0004-9514(14)60376-7

[B29] Willems TM Witvrouw E Delbaere K Mahieu N De Bourdeaudhuij I De Clercq D Intrinsic risk factors for inversion ankle sprains in male subjects: a prospective study Am J Sports Med 2005 33 3 415 423 10.1177/0363546504268137 15716258

[B30] Cross KM Worrell TW Leslie JE Van Veld KR The relationship between self-reported and clinical measures and the number of days to return to sport following acute lateral ankle sprains J Orthop Sports Phys Ther 2002 32 1 16 23 11787905 10.2519/jospt.2002.32.1.16

[B31] Denegar CR Hertel J Fonseca J The effect of lateral ankle sprain on dorsiflexion range of motion, posterior talar glide, and joint laxity J Orthop Sports Phys Ther 2002 32 4 166 173 11949665 10.2519/jospt.2002.32.4.166

[B32] van der Wees PJ Lenssen AF Hendriks EJ Stomp DJ Dekker J de Bie RA Effectiveness of exercise therapy and manual mobilisation in ankle sprain and functional instability: a systematic review Aust J Physiother 2006 52 1 27 37 16515420 10.1016/s0004-9514(06)70059-9

[B33] Pellow JE Brantingham JW The efficacy of adjusting the ankle in the treatment of subacute and chronic grade I and grade II ankle inversion sprains J Manipulative Physiol Ther 2001 24 1 17 24 10.1067/mmt.2001.112015 11174691

[B34] Whitman JM Childs JD Walker V The use of manipulation in a patient with an ankle sprain injury not responding to conventional management: a case report Man Ther 2005 10 3 224 231 10.1016/j.math.2004.10.003 16038858

[B35] Whitman JM Cleland JA Mintken PE Keirns M Bieniek ML Albin SR Magel J McPoil TG Predicting short-term response to thrust and nonthrust manipulation and exercise in patients post inversion ankle sprain J Orthop Sports Phys Ther 2009 39 3 188 200 19252260 10.2519/jospt.2009.2940

[B36] Springer BA Arciero RA Tenuta JJ Taylor DC A prospective study of modified Ottawa ankle rules in a military population Am J Sports Med 2000 28 6 864 868 11101110 10.1177/03635465000280061501

[B37] Gould N Repair of lateral ligament of ankle Foot Ankle 1987 8 1 55 58 3623363 10.1177/107110078700800111

[B38] Hollis JM Blasier RD Flahiff CM Simulated lateral ankle ligamentous injury. Change in ankle stability Am J Sports Med 1995 23 6 672 677 10.1177/036354659502300606 8600732

[B39] Staples OS Ruptures of the fibular collateral ligaments of the ankle. Result study of immediate surgical treatment J Bone Joint Surg Am 1975 57 1 101 107 804489

[B40] Brijker F Heijdra YF Van Den Elshout FJ Bosch FH Folgering HT Volumetric measurements of peripheral oedema in clinical conditions Clin Physiol 2000 20 1 56 61 10.1046/j.1365-2281.2000.00224.x 10651793

[B41] Martin RL Irrgang JJ Burdett RG Conti SF Van Swearingen JM Evidence of validity for the Foot and Ankle Ability Measure (FAAM) Foot Ankle Int 2005 26 11 968 983 16309613 10.1177/107110070502601113

[B42] Faul F Erdfelder E Lang AG Buchner A G*Power 3: a flexible statistical power analysis program for the social, behavioral, and biomedical sciences Behav Res Methods 2007 39 2 175 191 17695343 10.3758/bf03193146

[B43] Johnson EE Markolf KL The contribution of the anterior talofibular ligament to ankle laxity J Bone Joint Surg Am 1983 65 1 81 88 6848539

[B44] Landeros O Frost HM Higgins CC Post-traumatic anterior ankle instability Clin Orthop Relat Res 1968 56 169 178 10.1097/00003086-196801000-00019 5652775

[B45] Aradi AJ Wong J Walsh M The dimple sign of a ruptured lateral ligament of the ankle: brief report J Bone Joint Surg Br 1988 70 2 327 328 3126191 10.1302/0301-620X.70B2.3126191

[B46] Henschke N Boland RA Adams RD Responsiveness of two methods for measuring foot and ankle volume Foot Ankle Int 2006 27 10 826 832 17054886 10.1177/107110070602701013

[B47] Brodovicz KG McNaughton K Uemura N Meininger G Girman CJ Yale SH Reliability and feasibility of methods to quantitatively assess peripheral edema Clin Med Res 2009 7 1-2 21 31 10.3121/cmr.2009.819 19251582 PMC2705274

[B48] Childs JD Piva SR Fritz JM Responsiveness of the numeric pain rating scale in patients with low back pain Spine (Phila Pa 1976) 2005 30 11 1331 1334 15928561 10.1097/01.brs.0000164099.92112.29

[B49] Waddell G Newton M Henderson I Somerville D Main CJ A Fear-Avoidance Beliefs Questionnaire (FABQ) and the role of fear-avoidance beliefs in chronic low back pain and disability Pain 1993 52 2 157 168 10.1016/0304-3959(93)90127-B 8455963

[B50] Jacob T Baras M Zeev A Epstein L Low back pain: reliability of a set of pain measurement tools Arch Phys Med Rehabil 2001 82 6 735 742 10.1053/apmr.2001.22623 11387576

[B51] Crombez G Vlaeyen JW Heuts PH Lysens R Pain-related fear is more disabling than pain itself: evidence on the role of pain-related fear in chronic back pain disability Pain 1999 80 1-2 329 339 10.1016/S0304-3959(98)00229-2 10204746

[B52] Klenerman L Slade PD Stanley IM Pennie B Reilly JP Atchison LE Troup JD Rose MJ The prediction of chronicity in patients with an acute attack of low back pain in a general practice setting Spine 1995 20 4 478 484 10.1097/00007632-199502001-00012 7747233

[B53] Watson D Clark LA Tellegen A Development and validation of brief measures of positive and negative affect: the PANAS scales J Pers Soc Psychol 1988 54 6 1063 1070 10.1037/0022-3514.54.6.1063 3397865

[B54] Guyatt GH Feeny DH Patrick DL Measuring health-related quality of life Ann Intern Med 1993 118 8 622 629 8452328 10.7326/0003-4819-118-8-199304150-00009

[B55] Norkin CC White DJ Measurement of joint motion: a guide to goniometry 2003 2 Philadelphia: F.A. Davis

[B56] Elveru RA Rothstein JM Lamb RL Goniometric reliability in a clinical setting. Subtalar and ankle joint measurements Phys Ther 1988 68 5 672 677 3362980 10.1093/ptj/68.5.672

[B57] Jonson SR Gross MT Intraexaminer reliability, interexaminer reliability, and mean values for nine lower extremity skeletal measures in healthy naval midshipmen J Orthop Sports Phys Ther 1997 25 4 253 263 9083944 10.2519/jospt.1997.25.4.253

[B58] McPoil TG Cornwall MW The relationship between static lower extremity measurements and rearfoot motion during walking J Orthop Sports Phys Ther 1996 24 5 309 314 8902683 10.2519/jospt.1996.24.5.309

[B59] Kendall FP McCreary EK Provance PG Muscles, testing and function: with Posture and pain 1993 4 Baltimore, Md.: Williams & Wilkins

[B60] Van Dillen LR McDonnell MK Fleming DA Sahrmann SA Effect of knee and hip position on hip extension range of motion in individuals with and without low back pain J Orthop Sports Phys Ther 2000 30 6 307 316 10871142 10.2519/jospt.2000.30.6.307

[B61] Mulroy SJ Lassen KD Chambers SH Perry J The ability of male and female clinicians to effectively test knee extension strength using manual muscle testing J Orthop Sports Phys Ther 1997 26 4 192 199 9310910 10.2519/jospt.1997.26.4.192

[B62] Gribble PA Hertel J Denegar CR Chronic ankle instability and fatigue create proximal joint alterations during performance of the Star Excursion Balance Test Int J Sports Med 2007 28 3 236 242 10.1055/s-2006-924289 17447273

[B63] Hertel J Functional instability following lateral ankle sprain Sports Med 2000 29 5 361 371 10.2165/00007256-200029050-00005 10840868

[B64] Hertel J Braham RA Hale SA Olmsted-Kramer LC Simplifying the star excursion balance test: analyses of subjects with and without chronic ankle instability J Orthop Sports Phys Ther 2006 36 3 131 137 16596889 10.2519/jospt.2006.36.3.131

[B65] Kinzey SJ Armstrong CW The reliability of the star-excursion test in assessing dynamic balance J Orthop Sports Phys Ther 1998 27 5 356 360 9580895 10.2519/jospt.1998.27.5.356

[B66] Plisky PJ Rauh MJ Kaminski TW Underwood FB Star Excursion Balance Test as a predictor of lower extremity injury in high school basketball players J Orthop Sports Phys Ther 2006 36 12 911 919 10.2519/jospt.2006.2244 17193868

